# Longitudinal Association between Bullying Victimization and Depressive Symptoms in Chinese Early Adolescents: The Effect of Life Satisfaction

**DOI:** 10.1155/2024/6671415

**Published:** 2024-06-30

**Authors:** Yingying Tong, Faliang Xie, Xue Wen, Yonghan Li, Mengyuan Yuan, Xueying Zhang, Juan Chen, Gengfu Wang, Puyu Su

**Affiliations:** ^1^Department of Maternal, Child and Adolescent Health, School of Public Health, Anhui Medical University, No.81 Meishan Road, Hefei 230032, Anhui, China; ^2^Department of Environmental Medicine and Public Health, Icahn School of Medicine at Mount Sinai, New York 10029, NY, USA; ^3^Affiliated Psychological Hospital of Anhui Medical University, Anhui Mental Health Center, Fourth People's Hospital of Hefei, No.316 Huangshan Road, Hefei 230032, Anhui, China; ^4^Key Laboratory of Population Health Across Life Cycle, Ministry of Education of the People's Republic of China, Anhui Medical University, No 81 Meishan Road, Hefei 230032, Anhui, China; ^5^Anhui Provincial Key Laboratory of Population Health and Aristogenics, No 81 Meishan Road, Hefei 230032, Anhui, China

## Abstract

Bullying victimization is associated with an increased risk of depression among adolescents. However, few studies have examined the association between bullying victimization and depressive symptoms, the impact of particular dimensions of life satisfaction on this relationship, and whether these associations vary by gender. A multilevel model (MLM) was used to explore the relationship between 1,611 Chinese early adolescents' incidence of bullying victimization and depression and life satisfaction's effect on this relationship (60.5% boys, *M*_age_ = 12.48, SD = 0.48 at baseline). Bullying victimization was found to predict a high risk of depression in early adolescence. Further, we observed that life satisfaction mitigated the relationship between bullying victimization and depression; the MLM analysis indicated that these associations somewhat differed between genders. This study emphasized—based on ecological theory—four special dimensions of life satisfaction and bullying victimization in relation to depression risk. Additionally, this study provides novel insights into the correlation between bullying victimization and depression among Chinese early adolescents.

## 1. Introduction

Currently, depression is among the most common psychiatric disorders [1]. The estimated global prevalence of adolescent depression is 8% [2]. Specifically, early adolescence (aged 10–14 years)—a critical period for the onset of depression [3]—is characterized by rapid social and psychological changes, including increased socioemotional sensitivity and susceptibility to depression [4, 5]. During this developmental stage, adolescents begin experiencing more turbulent emotions than adults [6]. Changes in adolescents' nervous systems are accompanied by emotional changes, and they become extremely sensitive to negative emotions [7]. Depression imposes substantial personal, social, and economic burdens during this critical life transition, increasing adolescents' lifelong risk of impaired physical and mental health [2, 8, 9].

Bullying victimization refers to an individual's experience of being a target of unwanted aggressive behaviors enacted intentionally and repeatedly by another individual or group without being able to defend oneself owing to a power imbalance [10]. Ecological systems theory underscores that school is an important microsystem for—and is closely related to—adolescents' growth and development [11]. School bullying is an adverse life event associated with poor adjustment among adolescents [12]. A survey conducted in China revealed that 42.9% of students had been bullied by their peers, and of these, 34.7% had been bullied in school [13]. Research has widely indicated that bullying victimization is associated with varied adjustment difficulties including depression, anxiety, loneliness, social isolation, and even suicidal behavior [14, 15, 16]. Among these internalizing problems, depressive symptoms are a common mental health problem following bullying victimization. A meta-analysis suggested that victimized youths exhibit a greater risk of developing depressive symptoms than their nonvictimized peers [17].

Although previous research has identified bullying victimization's direct effect on depression, whether adolescent life satisfaction mitigates this effect remains unclear. Overall, low life satisfaction is a potent risk factor for depression [18, 19, 20]. Ecological theory provides an ideal framework to comprehensively understand how life satisfaction mitigates bullying victimization's effect on depression [11]. This theory can be summarized as a four-tiered structure of organizational risk and protective factors, which can subsequently inform targeted prevention strategies. The four levels—from micro to macro—are the individual, family, school, and societal levels. Since the 1990s, researchers have focused on specific dimensions of adolescent life satisfaction [21], such as satisfaction with family, peers, school, self, and the environment [22]. (1) The individual level includes factors such as gender, age, depression, bullying victimization, and satisfaction with oneself. (2) Regarding the family level, previous studies have demonstrated that the family environment and family education are important aspects of family satisfaction in early adolescence [23, 24, 25]. Notably, punitive education is a predominant method of parental education in China [26]. Negative interactions—including poor parenting, abuse, bullying victimization, and aggression—reduce family satisfaction, which, in turn, is strongly associated with the development of depressive symptoms among adolescents [27]. (3) Regarding the school level, the environment outside the family, especially the school context, becomes increasingly prominent [28]. Reportedly, school satisfaction—as a critical aspect of adolescent well-being [29]—contributes to school engagement [30]. Chinese culture is rooted in Confucianism and collectivism, which advocate maintaining social harmony, interdependence, obedience to authority, and compliance with group norms in interpersonal relationships [13]. (4) The social level: Severe friendship-related stress and undesirable teacher–student relationships reduce Chinese adolescents' satisfaction with school and peers; further, exposure to peer discrimination precipitates alienation and dissatisfaction with school, thus increasing their depressive symptoms [31]. However, these studies have focused only on a single dimension of life satisfaction. Accordingly, the socioecological framework helps us to understand the various dimensions of life satisfaction to mitigate bullying victimization's impact on depression—a necessary prerequisite for developing and implementing effective prevention and intervention measures.

Another vital factor for consideration during adolescent development is gender differences [32]. Depression tends to negatively affect girls more than boys in early adolescence [33]. Additionally, girls may experience more bullying-related psychosomatic effects than boys [34]. Biologically, girls exhibit greater activity in the left hemisphere and parietal lobe than boys; age-specific interactions in these regions are believed to reflect sex-specific changes during functional maturation [35, 36]. Additionally, specific areas of life—such as academic satisfaction, living standards, health, and relationships—exhibit gender differences among adolescents in terms of life satisfaction [37]. However, although female reports significantly greater levels of life satisfaction than male [33], they are more likely to suffer from depression. According to Becchetti and Conzo [38] women's life satisfaction is more affected by desirable or undesirable events or achievements—and they exhibit less resilience—compared to men.

This study used data from the Chinese Early Adolescent Cohort (CEAC) [39, 40], which is a cohort study to identify risk factors for mental and behavioral problems in early adolescence. Although some previous studies have examined bullying and depression [15, 17], to our knowledge, a clear research gap exists in exploring the interactions between bullying victimization and depressive symptoms, and life satisfaction's role in this relationship, especially in the context of Chinese adolescents. Additionally, ecological theory's application to the study of Chinese adolescents' mental health has been limited. To address this gap, our study employed a multilevel model (MLM) to explore the associations among bullying victimization, depression, and life satisfaction; notably, this study provides novel insights into the correlation between bullying victimization and depression among Chinese early adolescents.

## 2. Methods

The study protocol followed the guidelines of the Declaration of Helsinki and was approved by the ethical committee of Anhui Medical University (No. 20180083). Written informed consent was obtained from the participating students and their parents. Moreover, the Strengthening the Reporting of Observational Studies in Epidemiology (STROBE) statement was employed to report observational data (Table S1 in Supplementary Materials) [41].

### 2.1. Study Settings and Participants

This study's data were obtained from the CEAC [39], which was administered in a middle school in Huaibei City, Anhui Province, to explore and verify the risk factors for emotional and behavioral problems among adolescents; the CEAC was established in September 2019 using random cluster sampling. All enumerators were uniformly trained before the survey. The investigators were provided a self-administered questionnaire on adolescent mental health behaviors, which collected information regarding general demographic characteristics, such as gender, being an only child, self-assessment of family finances, and parents' educational levels; peer bullying; depression; and life satisfaction. During the survey, teachers in the students' classes maintained on-site discipline, investigators conducted rounds and answered doubts regarding the survey process, and the respondents were seated independently and completed the questionnaire autonomously. The questionnaire was completed and retrieved on the spot; the investigator quickly skimmed and verified the questionnaire content's completeness, and if any items were found to be missing, returned it to the participant to complete the questionnaire promptly. The inclusion criterion was as follows: seventh grade students enrolled in the September 2019 school year. The exclusion criteria were as follows: severe liver/kidney disease, physical disability, serious injury or infection, psychotropic drug use, and incomplete questionnaire data. All seventh grade students in the selected school were invited to participate in the baseline survey (time 1) after excluding students who did not fulfill the inclusion criterion. Follow-up surveys were administered in September 2020 (time 2) and September 2021 (time 3), during the COVID-19 pandemic. Finally, the data analysis included 1,611 students (Figure S1).

### 2.2. Study Protocol

The sample size was calculated according to the following formula:

(1) $$\begin{array}{c} n=\frac{Z_{1-\alpha /2}^{2}\left(1-P\right)}{\varepsilon^{2}P} \end{array}$$

According to a meta-analysis [42], the detection rate of depressive symptoms in middle school students is 28.4%; *α* = 0.05; confidence interval = 1˗*α* = 95%; relative error, *ε* = 10%; *n* ≈ 969. We finally included 1,611 participants, so our sample size is sufficient.

### 2.3. Depressive Symptoms

Self-reports of depressive symptoms across the four waves were assessed using the 20-item Chinese version of the Pediatric Depression Scale (CES-DC) [43]. This scale has been validated, with higher scores indicating more severe symptoms [8]. A cutoff score of 21 was considered as indicating a high probability of clinical depression. The scale has been widely used in Chinese adolescents and has shown ideal reliability [44] (Cronbach's *α* of 0.79; for correlations between CES-D scores at times 1, 2, and 3, see Table S2) [45].

### 2.4. Bullying Victimization

Questions regarding school bullying were adapted or modified from previous studies [10, 46]; two new items were added based on our social context. The responses are rated on a 5-point scale (1 = none, 2 = only once or twice, 3 = two–three times a month, 4 = approximately once a week, and 5 = several times a week). First, we provided the participants with a standard definition of bullying (qifu) in Chinese [47]. Subsequently, they were asked two questions regarding two parallel questions regarding how frequently they had bullied others or had been bullied in the past 2 months at school (details of specific items; see Table S3). Previous study has shown that the scale has high reliability and validity [48]. The *κ* values ranged from 0.85 to 0.96. Moreover, a consistency test was performed, resulting in a Cronbach's *α* ranging from 0.74 to 0.80.

### 2.5. Life Satisfaction

Participants' life satisfaction was rated on four dimensions—namely, home, school, peers, and self—using the Visual Mood Scale [49], which is useful for investigating life satisfaction [50]. Each dimension is evaluated on a scale ranging from 0 to 10, with “0” representing extremely dissatisfied and “10” representing extremely satisfied. The participants indicated their true feelings by marking the corresponding score across the four dimensions. The continuous values conformed to a normal distribution and were subsequently analyzed (Cronbach's *α* values were 0.76 for this study; for life satisfaction category endorsement at times 2 and 3, see Table S4).

### 2.6. Covariates

Considering previous studies [39, 46], the following covariates were included in the analysis: gender (girls or boys), age, perceived family economic status (low, medium, or high), relationship with parents (poor, medium, or good), number of friends, only child (yes or no), family type (nuclear, large, or single-parent), and body mass index.

### 2.7. Data Analysis

We aimed to assess the relative contribution of bullying victimization by using a sample of Chinese early adolescents on (a) concurrent measures of adolescent depression (baseline) and (b) future depression at two time points in adolescence (1 and 2 years postbaseline). An MLM was employed to examine whether adolescent life satisfaction exerted a unique effect on depressive symptoms during the first, second, and/or third waves. Depression observations were nested among the participants. Time was virtually coded to test the change from time 1 = 0 (i.e., as an intercept). The model included a random effect intercept specified by the participant, allowing for a change over time. Furthermore, at baseline and follow-up, we examined whether bullying victimization's effect on depressive symptoms depended on gender. Additionally, gender was included to determine whether life satisfaction's effect on the first, second, or third episodes of depressive symptoms varied (boys were coded as a reference). Finally, we assessed whether life satisfaction mitigated bullying victimization's effects on depressive symptoms. For secondary purposes, we included life satisfaction as a third variable to test whether the four dimensions of adolescent life satisfaction would mitigate school bullying's effect on depressive symptoms during waves two and/or three. We excluded participants who provided incomplete baseline data regarding bullying, life satisfaction, and the covariates. Multiple imputation (MI) was employed to estimate missing depression data during the second and third waves. Table S5 presents the details regarding the assignment of all variables. All analyses were performed using the Stata (version 14.2; Stata Corp, 2015), *R* studio (version 4.2.0.), and SPSS (version 27.0) softwares. Notably, *P* ≤ 0.05—a two-sided *P* value of 0.05 or less—was considered as indicating statistical significance.

## 3. Results

### 3.1. Sample Characteristics

The final analysis included 1,611 students, 60.50% of whom were boys. The response rate was 97.52%. At baseline, the participants' average age was 12.48 years (SD = 0.48), most of them (45.90%) were from a nuclear family, and 884 (52.40%) of them did not report depression.  Table 1 presents the participants' demographic information as well as their depression and bullying victimization scores.

### 3.2. Multilevel Model

#### 3.2.1. Adolescent Bullying Victimization, Gender, and Depressive Symptoms

Significant main effects on depression of bullying victimization (*β* = 1.62, *P* < 0.01), gender (*β* = 3.16, *P* < 0.01), and time (*P* < 0.01; Table 2) were observed. These results suggest that at all time points (waves), high bullying victimization levels were strongly associated with high depressive symptoms, and girls reported higher levels of depressive symptoms than boys.

**Figure 1:**
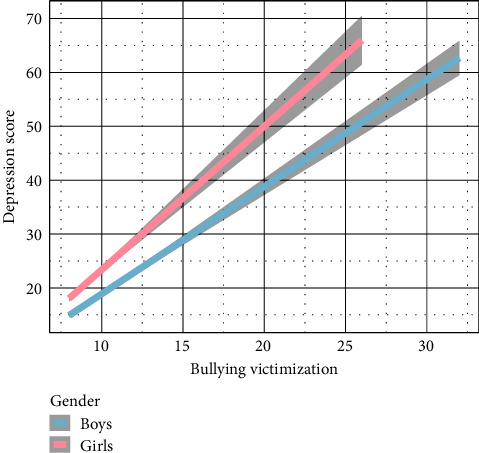
Depiction of the interaction between bullying victimization and gender predicting depressive symptoms.

**Figure 2:**
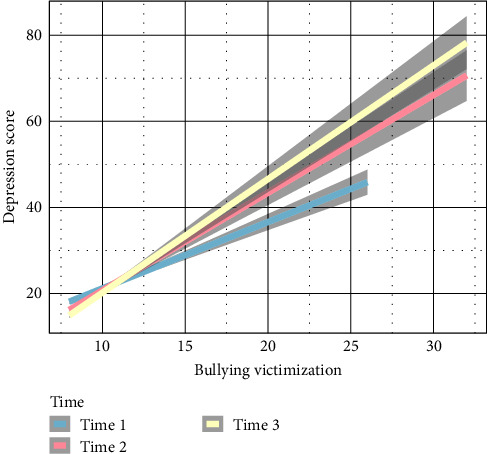
Depiction of the interaction between bullying victimization and time predicting depressive symptoms.

Along with the main effects of bullying victimization and gender, several important interactions were observed. Significant interactions were noted between bullying victimization and gender (*β* = 0.44, *P* = 0.01; Figure 1**)**. As shown in Figure 2, bullying victimization had a greater effect on depressive symptoms at time 1 versus 3, *β* = 0.61, *P* < 0.01, and at time 1 versus 2, *β* = 0.85, *P* < 0.01. Interestingly, there were significant three-way interactions among time and bullying victimization (for additional results; see Table 2 and Figure S2).

#### 3.2.2. Adolescent Bullying Victimization, Gender, Life Satisfaction, and Depressive Symptoms

When all four dimensions of life satisfaction were included in the same model, all exhibited significant main effects; moreover, a main effect of T2 life satisfaction on depressive symptoms was noted (family: *β* = −0.86, *P* < 0.01; friend: *β* = −1.04, *P* < 0.01; school: *β* = −0.65, *P* < 0.01, myself: *β* = −0.93, *P* < 0.01). Additionally, a main effect of T3 life satisfaction on depressive symptoms was observed (family: *β* = −0.96, *P* < 0.01; friend: *β* = −0.99, *P* < 0.01; school: *β* = −0.63, *P* < 0.01; myself: *β* = −1.10, *P* < 0.01). Gender's main effect was *β* = 2.16, *P* < 0.01, and bullying victimization's main effect remained significant, *β* = 0.95, *P* < 0.01. The interaction effects between bullying victimization and time (*P* < 0.01) and those between bullying victimization and gender (*β* = 0.33, *P* < 0.01) remained significant when life satisfaction was included in the model. Significant interactions were observed between adolescent bullying victimization and T2 or T3 life satisfaction and depressive symptoms (*P* < 0.01; for additional results, see Table 3). Significant three-way interactions were observed between time and bullying victimization (for the full results, see Table 3).

#### 3.2.3. Exploratory Analyses

Three-way interactions among bullying victimization, gender, and life satisfaction were explored. Several significant three-way interactions were observed (Table S6).

## 4. Discussion

This study systematically examined the longitudinal relevance of bullying victimization and depressive symptoms among Chinese adolescents. Overall, the MLM analysis indicated that bullying victimization among early adolescents influenced depression risk over the following 3 years and that this effect was stronger for girls than for boys. We extended previous research by demonstrating that all four dimensions of life satisfaction mitigate bullying victimization's effects on depression. This study offers novel insights into depression prevention among adolescents. Targeting life satisfaction in terms of its four dimensions—namely, individual, family, peers, and school—is a promising approach for improving depression among adolescents, particularly for those who have been bullied.

Bullying is a crucial social problem among children and adolescents that results in anxiety, depression, loneliness, reduced life satisfaction, and even self-harm [51, 52]. In our sample, 25.80%, 13.20%, and 11.20% of participants reported bullying at T1, T2, and T3, respectively. The prevalence of bullying has been decreasing, which may be related to the coronavirus disease 2019 (COVID-19). In the period of COVID-19, the Chinese government and education authorities recommend that schools mobilize students for home study and conduct online classes [53, 54], thus somewhat reducing offline interactions and communication among students, and thus reducing the prevalence of bullying. These rates are higher than those reported for bullying victims in the Youth Risk Behavior Survey (1.10%) [55]. The high prevalence of being bullied highlights the need to implement measures addressing bullying victimization among Chinese adolescents, especially early adolescents. Previous studies have reported a significant association between bullying victimization and depression [56, 57]. However, most of these were cross-sectional studies. This study adopted a cohort design and focused on the susceptibility period to depression. Being bullied is longitudinally correlated with an increased risk of depressive symptoms among adolescents. Numerous scholars have argued that depressive symptoms caused by bullying victimization among adolescents—if not treated timely—can even increase suicide risk [56, 57, 58, 59, 60].

Interestingly, our results revealed that the four specific dimensions of life satisfaction mitigated bullying victimization's effect on depression and may play a specific role in preventing depression. Bullying victimization is associated with not only adolescents' individual attributes but also their familial and social environment [56]. An ecosystem theory-based and applied cross-lagged panel network analysis reported that individual-level (gender, depression, and anxiety), family-level (child maltreatment), school-level (satisfaction with classmates), and societal-level (satisfaction with society) factors are associated with bullying victimization [56]. This emphasizes the need to consider both individual and environmental factors in preventing and controlling depression. Negative educational methods, such as physical punishment, name-calling, and blame, and incomplete family structures, such as left-behind children, seriously affect adolescents' mental health [26, 61]. In China, teachers are considered “temporary attachment figures” with safe haven functions [62], which may make adolescents feel cared, loved, and trusted, and can help reduce bullying victimization and subsequent negative emotions [63, 64]. Additionally, peer groups precipitate a sense of warmth, belonging, and connection, thus improving bullying victims' emotional adjustment and enhancing their subjective well-being [65]. Therefore, efforts to prevent and control depression can be initiated from the perspectives of preventing bullying victimization, integrating ecological theory, and implementing comprehensive interventions based on the four specific dimensions of life satisfaction. This highlights the importance of preventing and controlling depression as well as preventive measures' multifaceted nature and complexity.

In recent years, gender-based differences in depressive symptoms have been increasing [66]. Our study revealed that bullying victimization and the four dimensions of life satisfaction uniquely predict depressive symptoms in both genders in early adolescence—consistent with previous studies [67, 68]. Traditional gender stereotypes in Chinese society precipitate gender inequality, resulting in more severe depressive symptoms in female than in male adolescents [69]. Biologically, some studies have indicated sex-based differences in circulating testosterone levels and left inferior parietal lobule, middle temporal gyrus, calcarine, and right lingual gyrus thickness, whereas regional patterns of testosterone interactions may affect the understanding of gender-based differences in behavioral and adolescent-onset neuropsychiatric disorders [70]. The causes of depression risk among adolescents are complex and multifaceted, involving poor family/school environment and low family income [66]. Additionally, genetic factors are among the major risk factors for depression among adolescents [66]. Genetic and environmental sources of life satisfaction and depressive symptoms are typically shared, but the extent of influence varies between male and female adolescents, with less genetic contribution and a greater role of shared environmental influences in girls than in boys [68]. Future interventions should focus on this high-risk group of girls to prevent depression and bullying victimization among them.

### 4.1. Strength and Limitations

Our study's strengths are as follows: First, we designed and conducted a longitudinal study with seventh graders to investigate the association between bullying victimization and depression and explored the effect of the four dimensions of life satisfaction on this association. Second, this study had a high follow-up and response rate. Third, this study provides novel insights into the need for preventing depression and bullying victimization among adolescents from the perspective of the four dimensions of life satisfaction based on ecological systems theory. Simultaneously, our study has several limitations. First, this study's data were obtained through self-report questionnaires; hence, recall bias is possible. Second, the rating scale of life satisfaction was formulated referring to the visual analog scale, which includes four special dimensions of items related to Chinese adolescents' early development period. Future research can use more detailed life satisfaction scales to reduce bias. Third, the participants were early adolescents in Anhui Province, China; hence, these findings must be generalized to other populations with caution. Furthermore, cultural differences should be considered when exploring associations among gender, depressive symptoms, and life satisfaction in other countries. Fourth, our follow-up data was collected during the COVID-19 pandemic; however, the impact of COVID-19 on our study was not considered.

### 4.2. Implications

This study offers several practical implications. First, the prevalence of bullying victimization was found to be relatively high in this study, and exposure to bullying victimization increases the risk of depression among early adolescents, thus rendering the prevention of bullying in schools critical. Second, our study found that life satisfaction mitigates bullying victimization's effect on depression; therefore, enhancing adolescents' life satisfaction and well-being is a promising goal. For parents, maintaining a satisfactory parent–child relationship is crucial to enhancing adolescents' family satisfaction [26]. Schools should enhance students' satisfaction with school in various aspects, such as actively organizing fun psychological activities and promoting team communication [57]. We actively advocate home–school cooperation, which enhances adolescents' satisfaction with themselves and their friends, and the prevention of bullying and depression among adolescents more comprehensively.

This study provides valuable insights for future research. First, this study utilized data from early adolescents to conduct an MLM of risk factors (bullying victimization) and protective factors (life satisfaction) associated with depression; future research can focus on a wider range of special populations, including pregnant women, adults, and older adults. Second, our follow-up time was during adolescence itself, suggesting that future studies can extend the follow-up time to adulthood [71]. Future research can explore the mental health outcomes observed during adulthood among people exposed to bullying victimization in their early life course and identify more comprehensive preventive factors.

## 5. Conclusion

This study used an MLM to examine the relationship between bullying victimization and depression. This study's results suggest that bullying victimization among early adolescents predicts a greater risk of depression, especially among female adolescents. Life satisfaction mitigated bullying victimizations' effect on depression, and gender differences were also observed. This study's findings have important implications for preventing depression among Chinese adolescents: (1) The importance of enhancing adolescents' life satisfaction across various dimensions must be emphasized. (2) Bullying must be comprehensively prevented in schools by implementing joint interventions across multiple departments to effectively reduce depression among adolescents. (3) Female adolescents' mental well-being must be prioritized as a focal point.

## Figures and Tables

**Table 1:** Description of the sample characteristics.

Time 1 (*n* = 1, 611)	*N* (%)
Sex (male)	975 (60.50)
Only child (yes)	258 (16.00)
Family structure	
Nuclear	740 (45.90)
Large	253 (15.70)
Single-parent	596 (37.00)
Other	22 (1.40)
Self-perceived family economic status	
Low	182 (11.30)
Middle	1,199 (74.40)
High	230 (14.30)
Relationship with mother	
Poor	298 (18.50)
Medium	676 (42.00)
Good	637 (39.50)
Relationship with father	
Poor	475 (29.50)
Medium	669 (41.50)
Good	467 (29.00)
Relationship with teacher	
Poor	843 (52.30)
Medium	615 (38.20)
Good	153 (9.50)
Relationship with classmate	
Poor	504 (31.30)
Medium	795 (49.30)
Good	312 (19.40)
Number of friends	
<3	446 (27.70)
3–5	622 (38.60)
>5	543 (33.70)
Time 1	Mean (SD)
Age	12.48 (0.48)
Depression	20.90 (10.40)
BV	9.82 (2.64)
Time 2	Mean (SD)
Depression	18.44 (11.66)
BV	9.00 (2.05)
Time 3	Mean (SD)
Depression	17.21 (11.99)
BV	8.88(1.99)

*Note*. Time 1: September 2019; Time 2: September 2020; Time 3: September 2021. BV, bullying victimization.

**Table 2:** Adolescent bullying victimization and depressive symptoms.

IVs	*B*	*t* Value	*P* value
Time	—	**−7.39**	**<0.01**
BV	**1.62**	**25.31**	**<0.01**
Gender	**3.16**	**7.78**	**<0.01**
Time × BV	—	**9.24**	**<0.01**
Time 2 × BV	**0.85**	**7.06**	**<0.01**
Time 3 × BV	**0.61**	**8.74**	**<0.01**
Time × Gender	—	0.51	0.61
BV × Gender	**0.44**	**2.55**	**0.01**
Time × BV × Gender	—	**2.87**	**≤0.001**

*Note*. Bold values are statistically significant *P*  < 0.05 (*α* = 0.05). BV, bullying victimization.

**Table 3:** Adolescent bullying victimization and depressive symptoms.

IVs	*B*	*t* value	*P* value
Time	—	**−14.06**	**<0.01**
BV	**0.95**	**16.29**	**<0.01**
Gender	**2.16**	**6.68**	**<0.01**
T2 family satisfaction	**−0.86**	**−9.44**	**<0.01**
T2 myself satisfaction	**−0.93**	**−12.25**	**<0.01**
T2 school satisfaction	**−0.65**	**−7.68**	**<0.01**
T2 friend satisfaction	**−1.04**	**−11.68**	**<0.01**
T3 family satisfaction	**−0.96**	**−10.70**	**<0.01**
T3 myself satisfaction	**−1.10**	**−14.14**	**<0.01**
T3 school satisfaction	**−0.63**	**−8.12**	**<0.01**
T3 friend satisfaction	**−0.99**	**−10.43**	**<0.01**
BV × T2 family satisfaction	**0.11**	**5.32**	**<0.01**
BV × T2 myself satisfaction	**0.09**	**4.04**	**<0.01**
BV × T2 school satisfaction	**0.15**	**7.12**	**<0.01**
BV × T2 friend satisfaction	**0.11**	**4.16**	**<0.01**
BV × T3 family satisfaction	**0.14**	**5.76**	**<0.01**
BV ×T3 myself satisfaction	**0.11**	**4.98**	**<0.01**
BV × T3 school satisfaction	**0.10**	**4.43**	**<0.01**
BV × T3 friend satisfaction	**0.10**	**5.66**	**<0.01**
Time × BV	—	**5.91**	**<0.01**
T2 × BV	**0.41**	**3.57**	**<0.01**
T3 × BV	**0.32**	**5.19**	**<0.01**
Time × Gender	—	0.23	0.82
BV × Gender	**0.33**	**2.16**	**0.03**
Time × BV × Gender	—	**3.89**	**<0.01**

*Note*. Bold values are statistically significant *P* < 0.05 (*α* = 0.05). T2, Time 2; T3, Time 3; BV, bullying victimization.

## Data Availability

The data that support the findings of this study are available on request from the corresponding author. The data are not publicly available due to privacy or ethical restrictions.

## Supplementary Materials

Supplementary Materials Table S1: shows that the STROBE Statement checklist, detailing items to be included in reports of observational studies. Table S2: shows that the correlations between CES-D scores across three different time points (*Note*. *⁣*^*∗∗∗*^*p* < 0.01). Table S3: shows that two questions regarding two parallel questions regarding how frequently they had bullied others or had been bullied in the past two months at school. Table S4: shows that the distribution of life satisfaction categories at the second and third time points.: Table S5 shows that the assignment of variables in the study (*Note*. BMI is body mass index). Table S6: shows that the relationship between adolescent bullying victimization, gender, and depressive symptoms. Figure S1: shows that the study follow chart. Figure S2: shows that depiction of the interaction between time and gender predicting depressive symptoms.
