# Supplementary material for: Longitudinal Association between Bullying Victimization and Depressive Symptoms in Chinese Early Adolescents: The Effect of Life Satisfaction
Source: Depress Anxiety. 2024 Jun 30;2024:6671415. doi: 10.1155/2024/6671415 (PMC11918514; doi:10.1155/2024/6671415)
Supplement: Supplementary Materials — Table S1: shows that the STROBE Statement checklist, detailing items to be included in reports of observational studies. Table S2: shows that the correlations between CES-D scores across three different time points (Note. ⁣∗∗∗p < 0.01). Table S3: shows that two questions regarding two parallel questions regarding how frequently they had bullied others or had been bullied in the past two months at school. Table S4: shows that the distribution of life satisfaction categories at the second and third time points.: Table S5 shows that the assignment of variables in the study (Note. BMI is body mass index). Table S6: shows that the relationship between adolescent bullying victimization, gender, and depressive symptoms. Figure S1: shows that the study follow chart. Figure S2: shows that depiction of the interaction between time and gender predicting depressive symptoms. [file 6671415.f1.docx]

**Supplementary materials**

**Table S1.** STROBE Statement-checklist of items that should be included in reports of observational studies.

**Table S2.** Correlations between CES-D scores at times 1, 2, and 3.

**Table S3.** Detailed information for school bullying.

**Table S4.** Life satisfaction category endorsement at times 2 and 3.

**Table S5.** Assignment of the variables.

**Table S6.** Adolescent bullying victimization, gender and depressive symptoms.

**Fig. S1.** The study follow chart.

**Fig. S2.** Depiction of the interaction between time and gender predicting depressive symptoms.

**Table S1.** STROBE Statement-checklist of items that should be included in reports of observational studies.

|  | | Item No. | Recommendation | | Page  No. |  | |  |  |
| --- | --- | --- | --- | --- | --- | --- | --- | --- | --- |
| **Title and abstract** | | 1 | (*a*) Indicate the study’s design with a commonly used term in the title or the abstract | | 1, 3 |  | |  |  |
|  |  |  | (*b*) Provide in the abstract an informative and balanced summary of what was done and what was found | | 3 |  | |  |  |
| Introduction | | | | | |  | |  |  |
| Background/rationale | | 2 | Explain the scientific background and rationale for the investigation being reported | | 4-8 |  | |  |  |
| Objectives | | 3 | State specific objectives, including any prespecified hypotheses | | 8 |  | |  |  |
| Methods | | | | | |  | |  |  |
| Study design | | 4 | Present key elements of study design early in the paper | | 8-9 |  | |  |  |
| Setting | | 5 | Describe the setting, locations, and relevant dates, including periods of recruitment, exposure, follow-up, and data collection | | 8-9 |  | |  |  |
| Participants | | 6 | (*a*) *Cohort study*—Give the eligibility criteria, and the sources and methods of selection of participants. Describe methods of follow-up  *Case-control study*—Give the eligibility criteria, and the sources and methods of case ascertainment and control selection. Give the rationale for the choice of cases and controls  *Cross-sectional study*—Give the eligibility criteria, and the sources and methods of selection of participants | | 12-13 |  | |  |  |
|  |  |  | (*b*) *Cohort study*—For matched studies, give matching criteria and number of exposed and unexposed  *Case-control study*—For matched studies, give matching criteria and the number of controls per case | | 12-13 |  | |  |  |
| Variables | | 7 | Clearly define all outcomes, exposures, predictors, potential confounders, and effect modifiers. Give diagnostic criteria, if applicable | | 9-11 |  | |  |  |
| Data sources/ measurement | | 8* | For each variable of interest, give sources of data and details of methods of assessment (measurement). Describe comparability of assessment methods if there is more than one group | | 9-11 |  | |  |  |
| Bias | | 9 | Describe any efforts to address potential sources of bias | | Not reported |  | |  |  |
| Study size | | 10 | Explain how the study size was arrived at | | Not reported |  | |  |  |
| Quantitative variables | | 11 | | Explain how quantitative variables were handled in the analyses. If applicable, describe which groupings were chosen and why | 8-9 | |  | |  |
| Statistical methods | | 12 | | (*a*) Describe all statistical methods, including those used to control for confounding | 12-13 | |  | |  |
|  |  |  |  | (*b*) Describe any methods used to examine subgroups and interactions | 12-13 | |  | |  |
|  |  |  |  | (*c*) Explain how missing data were addressed | 12-13 | |  | |  |
|  |  |  |  | (*d*) *Cohort study*—If applicable, explain how loss to follow-up was addressed  *Case-control study*—If applicable, explain how matching of cases and controls was addressed  *Cross-sectional study*—If applicable, describe analytical methods taking account of sampling strategy | 12-13 | |  | |  |
|  |  |  |  | (*e*) Describe any sensitivity analyses | Not reported | |  | |  |
| Results | | | | | | | | |  |
| Participants | | 13* | | (a) Report numbers of individuals at each stage of study—eg numbers potentially eligible, examined for eligibility, confirmed eligible, included in the study, completing follow-up, and analysed | Figure S1 | |  | |  |
|  |  |  |  | (b) Give reasons for non-participation at each stage | Figure S1 | |  | |  |
|  |  |  |  | (c) Consider use of a flow diagram | Figure S1 | |  | |  |
| Descriptive data | | 14* | | (a) Give characteristics of study participants (eg demographic, clinical, social) and information on exposures and potential confounders | 13 | |  | |  |
|  |  |  |  | (b) Indicate number of participants with missing data for each variable of interest | Figure S1 | |  | |  |
|  |  |  |  | (c) *Cohort study*—Summarise follow-up time (eg, average and total amount) | 13-20 | |  | |  |
| Outcome data | | 15* | | *Cohort study*—Report numbers of outcome events or summary measures over time | 13-20 | |  | |  |
|  |  |  |  | *Case-control study—*Report numbers in each exposure category, or summary measures of exposure |  | |  | |  |
|  |  |  |  | *Cross-sectional study—*Report numbers of outcome events or summary measures |  | |  | |  |
| Main results | | 16 | | (*a*) Give unadjusted estimates and, if applicable, confounder-adjusted estimates and their precision (eg, 95% confidence interval). Make clear which confounders were adjusted for and why they were included | Not reported | |  | |  |
|  |  |  |  | (*b*) Report category boundaries when continuous variables were categorized | 10 | |  | |  |
|  |  |  |  | (*c*) If relevant, consider translating estimates of relative risk into absolute risk for a meaningful time period | Not reported | |  | |  |
| Discussion | | | | | | |  |  |  |
| Key results | 18 | | | Summarise key results with reference to study objectives | 21-22 | |  | | |
| Limitations | 19 | | | Discuss limitations of the study, taking into account sources of potential bias or imprecision. Discuss both direction and magnitude of any potential bias | 24-25 | |  | | |
| Interpretation | 20 | | | Give a cautious overall interpretation of results considering objectives, limitations, multiplicity of analyses, results from similar studies, and other relevant evidence | 22-24 | |  | | |
| Generalisability | 21 | | | Discuss the generalisability (external validity) of the study results | 25-26 | |  | | |
| Other information | | | |  | | |  |  |  |
| Funding | 22 | | | Give the source of funding and the role of the funders for the present study and, if applicable, for the original study on which the present article is based | 27 | |  | | |

*Give information separately for cases and controls in case-control studies and, if applicable, for exposed and unexposed groups in cohort and cross-sectional studies.

**Table S2.** Correlations between CES-D scores at times 1, 2, and 3.

| Pairwise cases | | | |
| --- | --- | --- | --- |
|  | Time 1 (Wave I) | Time 2 (Wave III) | Time 3 (Wave IV) |
| Time 1 | - |  |  |
| Time 2 | .529^***^ | - |  |
| Time 3 | .383^***^ | .601^***^ | - |

***Note*.** ^***^*p* <0 .001

**Table S3.** Detailed information for bullying victimization.

| Variables | Items | Categories |
| --- | --- | --- |
| **Bullying victimization** |  | 1=none, 2=1-2 times a week, 3=3-5 times a week, 4=6 or more times a week |
| Verbal | Other students give me insulting nicknames, call me names, or make fun of and criticize me.  Other students give me insulting nicknames because I speak with a different accent than they do. |  |
| Relational | Other students spread rumors about me and try to make other people dislike me.  Other students keep me out of certain activities, exclude me from their friends, or make their friends ignore me. |  |
| Physical | Other students have hit, kicked, pushed, slammed, grounded or threatened me.  Other students forced me to ask for money, or took or damaged my things. |  |
| Cyber | Other students have used the Internet or cell phone text messages to call me names, insult me, or threaten me.  Other students have used the telephone to call me names, insult me, or threaten me. |  |

**Table S4.** Life satisfaction category endorsement at times 2 and 3.

| **Life satisfaction category** | **Time 2** | **Time 3** |
| --- | --- | --- |
| Family | 8.49(2.02) | 8.37(2.11) |
| Friend | 7.92(2.10) | 7.91(1.98) |
| School | 7.78(2.22) | 6.96(2.63) |
| Myself | 6.71(2.39) | 7.12(2.31) |

**Table S5.** Assignment of the variables.

| **Variables** | **Items** | **Types** | **Variable assignment** |
| --- | --- | --- | --- |
| Gender | Gender? (male or female) | categorical | 1 = male; 2 = female |
| Age | Calculated by the date of birth | numerical |  |
| Only child | Are you the only child in your family? | categorical | 0 = no; 1 = yes |
| Family type | What’s your family structure like? | categorical | 1 = nuclear family  2 = single-parent  family;  3 = extended family;  4 = others |
| Family economic | How about your family financial status? | categorical | 1= poor; 2 = medium; 3 = good |
| Academic performance | How about your academic performance in your class? | categorical | 1 = poor; 2 = general; 3 = good |
| Relationship with mother | How is your relationship with ordinal your mother? | categorical | 1 = poor; 2 = general;3 = good |
| Relationship with father | How is your relationship with ordinal your mother? | categorical | 1 = poor; 2 = general; 3 = good |
| Relationship with teacher | How is your relationship with your teachers? | categorical | 1 = poor; 2 = general; 3 = good |
| Relationship with classmates | How is your relationship you’re your classmates? | categorical | 1 = poor; 2 = general; 3 = good |
| Number of friends | How many friends you can depend on? | categorical | 1 = less than 3 friends;  2 = 3 to 5 friends;  3 = more than 5 friends |
| BMI | Calculated using height and weight data of participants | numerical | - |
| Depression | CES-DC (The total score of the 20 items was 60, and a score above 20 indicated the presence of depressive symptoms. (Li, Zhu, 2018). | numerical | - |
| Life satisfaction | Visual analogue life satisfaction scale scores. Assessed across four dimensions: family, society, school, and self-assessed, with score range from 0 to 10. | numerical | - |
| Bullying victimization | Contains 8 items referencing to Zhang et al. (2019) | numerical | - |

***Note.*** BMI is body mass index.

**Table S6.** Adolescent bullying victimization, gender and depressive symptoms.

| IVs | B | t-Value | p-Value |
| --- | --- | --- | --- |
| Gender × BV × Family | **-** | **3.00** | **0.01** |
| Gender × BV × Friend | **-** | **2.12** | **0.03** |
| Gender × BV × School | **-** | **2.22** | **0.03** |
| Gender × BV × Myself | - | 0.93 | 0.36 |

***Notes:*** Bold values are statistically significant *p* <0 .05 (alpha=0.05); BV, Bullying victimization.

Recruitment in a middle school in Huaibei city, Anhui Province, China

N = 1860

Excluded n = 46

Refuse to participate (n = 46)

Baseline (Wave 1. Sep 2019), eligible participant n=1814

bullying victimization scale (n=1784)

Excluded n=63

Refuse to participate (n = 23)

Transfer to other school (n = 40)

1. year follow-up (Wave 2. Sep 2020),

n=1751 enrolled

Excluded n=96

Refuse to participate (n = 25)

Transfer to other school (n = 71)

1. year follow-up (Wave 3. Sep 2021),

n=1655 enrolled

bullying victimization scale (n=1658)

Missing data for bullying victimization and confounding variables (n=44)

A total of 1,611 participants included in all waves

**Figure S1.** The study follow chart.

15

17

19

21

23

1.0

1.5

2.0

2.5

3.0

Time

Depression score

Gender

Boys

Girls

**Fig. S2.** Depiction of the interaction between time and gender predicting depressive symptoms.
